# A Survey of Calf Housing Facilities Pre-Weaning, Management Practices and Farmer Perceptions of Calf Welfare on Irish Dairy Farms

**DOI:** 10.3390/ani13061019

**Published:** 2023-03-10

**Authors:** Alison M. Sinnott, Eddie A. M. Bokkers, John Paul Murphy, Emer Kennedy

**Affiliations:** 1 Teagasc, Animal and Grassland Research and Innovation Centre, Moorepark, Co. Cork, P61 P302 Fermoy, Ireland; 2 Animal Production Systems Group, Wageningen University & Research, P.O. Box 338, 6700 AH Wageningen, The Netherlands

**Keywords:** calf housing, calf welfare, farmer welfare, calf management systems, labour efficiency, alternative housing, dairy calf, Irish agriculture

## Abstract

**Simple Summary:**

Rearing facilities directly affect the viability and suitability of calf rearing systems on-farm; however, it is unknown whether facilities in the Republic of Ireland are fit for purpose, or sufficiently consider calf and farmer welfare. Current housing facilities and management practices on Irish farms were reviewed to determine if calves are reared in appropriate facilities with management decisions that safeguard calf and farmer welfare. Fifty-one farms in the Munster region of the Republic of Ireland were visited twice: (1) Pre-calving (December–January; farmer interview and housing structural evaluation); (2) During peak calving (January–March; short farmer interview and housing environmental evaluation). The results showed farmers planned sufficiently for spring, providing appropriate housing and space allowances for calves, facilitating group housing post-birth, influencing calf welfare positively. On-farm housing that was not purposely built for rearing calves (e.g., straw storage) is frequently converted to be used to rear calves, meaning sheds must be modified to appropriately cater for calf needs (e.g., drainage and slopes to facilitate cleaning out). Management components of rearing systems appear in line with current recommendations (e.g., advice against feeding waste milk), however some areas require attention (e.g., colostrum testing and extended working hours), to safeguard calf welfare and reduce associated workloads. In conclusion, housing provisions are sufficient for calf numbers born in Ireland, however housing and management should be continually reviewed to consider calf and farmer welfare.

**Abstract:**

It is unknown whether calf rearing facilities in the Republic of Ireland are fit for purpose, or if facilities sufficiently consider calf and farmer welfare. The aim of this study was to review current calf housing facilities and management practices on Irish farms to determine if calves are reared in structurally appropriate facilities with management decisions that safeguard calf and farmer welfare. Fifty-one farms located in the Munster region in the Republic of Ireland were visited twice: (1) Pre-calving (December–January) and (2) During peak calving (January–March). During visit one, herd owners completed a questionnaire regarding calf housing and management practices on-farm and each facility used to rear calves was measured (measurement of cubic air capacity, ventilation, pen area, drainage etc.) without calves being present. Visit two consisted of a short interview with the principal calf manager to validate previously asked questions and environmental based measurements of each calf house that had been recorded, with any deviation from the first visit noted (measurements of temperature, wind speed, light intensity, facility provisions in-house and in-pen; calves present). Average herd size was 254, operating a spring calving system with a median calving season length of 11.6 weeks. While most farms expanded (88%; N = 51), this did not appear to have negatively affected calf space allowances (9.9% houses overcrowded at a space allowance of 1.5 m^2^/calf; N = 121). Calves were most commonly housed in group sizes of <12 (71.6% of all groupings; N = 394), with farmers moving away from individual housing for a period immediately post-birth, to grouping them immediately instead (58.8%; N = 51). The number of farmers testing colostrum was 31.4% (N = 51). Although the calving season was compact, most farmers were unconcerned about the upcoming spring workload (58.8%; N = 51). Farms appeared sufficiently prepared for spring, with most using the same number or less sheds during visit two than declared in visit one (76.5%; N = 51). To conclude, farmers made sufficient provision for calf housing and space allowances for calves that facilitated group housing post-birth. While structural and management components of rearing systems appear in line with sectoral recommendations, certain areas require attention on many farms (e.g., colostrum testing) to safeguard calf welfare and reduce the workload associated with calf rearing for farmers.

## 1. Introduction

Post-quota expansion has changed the dynamic of the Irish dairy sector in recent years, whereby herd size [1] and the number of new entrants into dairy farming [2] have increased, leading to a greater volume of milk production (increased by 41% from 2015 to 2019; [3]). Increases in herd size understandably result in more calves being born on-farm annually. However, any increases in herd size should not lead to unsustainable workloads and poor calf welfare created by sub-optimal management practices and sub-standard facilities.

In Ireland, calf housing standards are regulated under the council of the European Union (2008/119/EC), which details the minimum specifications required (e.g., space requirement of 1.5 m^2^ for calves <150 kg/<19 weeks; [4]). However, in this generalized framework, considerably varied housing systems can be implemented while still complying with outlined regulations [5]. Although Barry et al. [6] identified Irish farms as providing sufficient space allowance, the states of other facility factors are unknown. Similarly, while Brown et al. [7] examined calf housing design on commercial farms in Northern Ireland, calving patterns and subsequent housing requirements vary greatly to the south of Ireland (year round calving vs. compact seasonal spring calving). Consequently, it is largely unknown if current calf rearing facilities in the Republic of Ireland are fit for purpose and whether facilities have grown in line with herd expansion and are of sufficient quality.

Structural components of indoor housing can have negative implications on calf welfare. For example, inappropriate drainage can cause excrement build-up followed by increased bacterial growth, eventually leading to health complications in calves [8]. This might happen when pre-existing sheds are converted for the purpose of calf rearing. Such structures are often only suited for temporary use and eventually require a purpose-built facility [9]. Previous research has echoed this, showing that calves are at a higher risk of severe respiratory disease in such facilities (comparison of mono-pitch, Patterson, kennel, climatic, converted and mechanically ventilated housing; [10]). Although structural elements of calf housing influence welfare, the management decisions and practices employed on-farm also play an integral role. For example, calves kept in smaller group sizes (<10 calves) had less respiratory issues than groups of 12–18 calves [11]. Appropriate airspace (at least 7 m^3^/calf) and prohibiting shared airspace with older stock helps prohibit respiratory infections [12]. Additionally, greater floor space increases the opportunity for active behaviours (i.e., social interactions, playing and walking), likely satisfying needs to perform a normal range of these behaviours [13]. In addition to ensuring appropriate environments promote calf welfare, it is important to also consider the needs of the calf carer. Good housing systems should facilitate the efficient completion of routine tasks associated with calf care [14]. Such provisions include accessible pens for inspection and care of sick calves [14] as well as tractor access to allow the cleaning out of rearing pens [15]. To ensure calf rearing progresses in a viable and sustainable way, the needs of both calf and carer should be considered collectively to encompass a One Welfare approach, to further develop farm management practices (Pinillos, 2018).

Therefore, the aim of this study was to review current calf housing facilities and management practices on Irish farms in the Munster region to determine if calves are reared in structurally appropriate facilities, with management decisions that safeguard calf welfare. Additionally, this research aimed to evaluate if appropriate measures are in place to promote labour efficient practices for calf carers.

## 2. Material and Methods

This study was carried out between 9 December 2019 and 13 March 2020. Ethical approval for the study was received from the Teagasc animal ethics committee (TAEC241-2019).

### 2.1. Herd Selection

This study focused solely on herds where the predominant enterprise was dairy (based on herd size, active engagement with dairy related knowledge transfer and technologies). Of 51 farms surveyed, 88.2% operated a dairy-only enterprise and 11.8% operated a dairy-mixed enterprise: four dairy-beef, one dairy-beef-tillage, one dairy-poultry. Suitable herds were convenience sampled and located within approximately 80 km of Teagasc Moorepark to facilitate data collection and minimize associated travel. This area represents the region most densely populated with dairy herds in Ireland, with over 56% of dairy cows in the Republic of Ireland located in it [16]. It must be acknowledged however, that this study population is not representative of the whole country of Ireland. To reflect current dairy systems in Ireland, herds that adopted a predominately spring calving system (February–April; calving >90% of the herd in this timeframe) were sought. A minimum herd size threshold of 75 cows was imposed (average herd size in Ireland at the time). In addition, herds had to be subscribed to HerdPlus (data management and reporting system operated by the Irish Cattle Breeding Federation; ICBF, Bandon, Co. Cork, Ireland) while also actively participating in a Teagasc discussion group. The animals in the herds used in this study were mainly Holstein Friesian and Holstein Friesian × Jersey, which is representative of the Irish national dairy herd [17]. Herds were selected by contacting discussion group facilitators, with facilitators inviting each group member (herd owner) to participate. Potential participants were informed of study objectives, measurements to be undertaken, information they were required to supply and feedback they would receive after study completion. Expressions of interest for inclusion were collected by the facilitator and a list of willing participants and associated contact details was compiled. Fifty-two herd owners agreed to partake (74.3% response rate), with herd sizes ranging from 87 to 550 cows; herd owners were contacted by telephone for specific farm location and a suitable visit time and date.

### 2.2. Survey Composition

Each farm was visited twice; the first was conducted pre-calving between 9 December 2019 and 17 January 2020. During this visit, herd owners completed a comprehensive face-to-face questionnaire regarding calf housing and management practices on-farm (survey available upon request). It contained 81 questions (2 open-ended questions; 79 closed questions) categorized into eight sections. Section one focused on general information of the farm enterprise such as number of cows expected to calve, date calving expected to commence, expected date of peak calving. Section two sought farmer opinions, asking questions on a range of topics; calf welfare on their farm, calf husbandry skills, concerns and how prepared they are for the upcoming calving season (rank from 1–10). Section three focused on housing facilities for calves on the farm, providing information on where calves were reared (indoor, outdoor or both), how many houses are used to rear calves, whether they are purpose built, when they were last updated, and the farmer’s plans to invest in calf rearing facilities in future. Section four asked questions regarding calving and first feeding (i.e., number of labour units on-farm, number of people involved in calf rearing and colostrum feeding and management). Section five gathered information on post-colostrum feeding practices. Section six related to animal health and antibiotic usage. Section seven asked about calf rearing hygiene practices. Finally, section eight focused on weaning and the sale of calves. Physical measurements pertaining to calf housing facilities were also gathered by the research team.

The second visit (23 January to 13 March 2020) aimed to coincide with peak calving (based on information farmers supplied during the first visit). Visit two consisted of a short interview with the principal calf manager to validate questions asked during visit one. There were seven questions regarding the number of cows to calve, the date calving started, how many cows had calved up to the point of visit, plans for the sale of calves and calf health. Environmental based measurements of each calf housing facility were also gathered by the research team. One herd owner declined a second visit.

Full consent was given by participants, through signing a GDPR data release form, to allow collection of data and analyses of their data on a group basis. For each farm visit, two people were present: an individual experienced in animal handling and conducting housing and environmental measurements and an individual who had received basic training in the aforementioned.

### 2.3. On-Farm Measurements

#### 2.3.1. Facility Measurements

During the first farm visit, each facility used to rear calves was measured (reducing the time required on-farm during visit two). No calves were present at the time of facility measurements. To determine cubic air capacity of the building the length, width, height at ridge and height at eaves were taken using a laser distance meter (Spectra Precision QM95 Laser Distance meter—range 0–200 m; Trimble Navigation Ltd. Dayton, OH, USA).

Within each calf house, facility measurements were taken of all group and individual pens using the laser distance meter. The total area of each pen was calculated to determine the number of calves each pen could accommodate (based on a minimum space requirement of 1.5 m^2^; EU, 2008). Drainage conditions were considered by measuring the slope of the floor and noting the type of drainage present (i.e., channel or free flow). Slope was measured by placing a spirit-level in the middle of the unobstructed floor surface. Using a clinometer application (Plain Code Clinometer; validated by manual measurement of slope) on a Samsung Galaxy S7 mobile phone, the phone was placed on the spirit level and the slope figure was presented digitally. If more than one slope was present in the surrounding area, all slopes were recorded. A trundle wheel (DuraWheel DW-PRO Distance Measuring Wheel 12.5 Diameter; DuraWheel, Pennsburg, PA, USA) was used to measure the distance of the calving pen to the calf house.

#### 2.3.2. Environmental Measurements

During the second visit, environmental based measurements were taken in each calf house, as well as area measurements taken during the first visit were checked. If the calf housing plan deviated from the first visit, it was noted. If any additional houses were in-use for calves, both facility and environmental measurements were taken.

Measurements of wind speed, temperature, relative humidity, and light intensity was recorded from both the internal and external environments. To measure wind speed, an anemometer (Kestrel 1000 Wind Meter; Kestrel Meters, Boothwyn, PA, USA) was used in three different positions at chest height; (i) vertically parallel with measurer, (ii) vertically perpendicular to the measurer and (iii) horizontal to the measurer. The same three measurements were taken externally, outside of the house, in an area free from obstructions. Internal house measurements were taken at the mid-point of each house (regardless of whether it was in a pen or alleyway). The anemometer was held in each position to give a more accurate representation of average wind speed from all directions. The average and maximum figure was recorded for each position.

Temperature and relative humidity were measured using a data logger (Tinytag TGP 4017 Temperature Data Logger; Tinytag, Chichester, UK). This recorded measurements constantly at one-minute intervals. One data logger was placed outside the house, and one was placed inside, in areas free from obstructions for the duration of data collection. A timestamp was recorded for each device upon entry and exit of the house, so data was comparable (minimum measurement duration was five minutes).

Light intensity (LUX) was measured using the Doggo Apps Lux Light Meter application [18,19] on a Samsung Galaxy S7 mobile phone. The phone was held horizontally to the sun (externally), and horizontally to the roof of the house (internally), without any obstruction of light. One measurement was taken in each area (internally at the centre point of the house and externally), 50 cm (approximate calf height) from ground level. The maximum figure for natural lux from each individual measurement was recorded.

A map was made of the house including each pen and the number of calves housed within. This map provided a reference for each house, whereby actual space allowance per calf could be calculated using previous measurements of pens. Pens were selected at random upon entering the house. The presence of water, concentrates, and availability and type of forage was noted for all pens within the house. Two measurements of wind-speed were taken in each selected pen at calf height (50 cm from bedding level). In addition, environmental based measurements were recorded using five pens in each building (based on a sample size calculation).

### 2.4. Data Editing

To facilitate comparisons among farms, continuous data related to herd size and percentage rate of expansion (relative to pre-quota abolition cow numbers) were converted to categorical data. For herd size, 1 = <150 cows, 2 = 151–200 cows, 3 = 201–300 cows and 4 = >300 cows (based on quartiles). For percentage rate of expansion, 1 = <10%, 2 = 11–20%, 3 = 21–35% and 4 = >35% (based on quartiles). For a number of questions asked, farmers could select multiple responses. As such, for responses where this occurred, total population numbers exceed N = 51 (total number of farmers surveyed). Farmer opinion related questions were scored from 1 to 10; however, this was collapsed into a six-point scale where 1 = 1, 2 = 2 and 3, 3 = 4 and 5, 4 = 6 and 7, 5 = 8 and 9 and 6 = 10. Responses for 1 and 10 were allocated their own point as these were the two extremes of responses (i.e., no possibility outcome could be better or worse, whereas if 2 or 9 were included outcome could improve or deteriorate further). Additionally, for ease of interpretation and to facilitate word association, each point was assigned a description ranging from one extreme to the other (e.g., extremely unconcerned, very unconcerned, somewhat unconcerned, somewhat concerned, very concerned, and extremely concerned). While we acknowledge pitfalls in collapsing data after collection, in this instance it facilitates data interpretation, and Colvin and Gorgun [20] highlighted that values are largely unaffected by collapsing data following collection).

Space allowance provided was calculated for individual houses by summing the total area available to calves, divided by the number of calves present in the house during evaluation. This figure was compared independently to the following space allowances: 1.5 m^2^ (minimum space allowance requirement; [21]), 1.7 m^2^ (lower space allowance recommendation; [14]) and 2.0 m^2^ (upper space allowance recommendation; [12]). Cubic air capacity (length × width × height of house) was calculated by dividing the total cubic volume of a house by 7 m^3^. Where house height differed at the eave and ridge, the average of these two figures is used in the calculation. This provided a figure for the total number of calves a house can hold, which was compared to the number of calves actually in the house during evaluation. If a house did not provide sufficient space allowance or cubic air capacity for the calves housed within, it was recorded as overcrowded.

### 2.5. Statistical Analysis

Statistical analyses were conducted using SAS software (Version 9.4, SAS Institute Inc. (Cary, NC, USA), 2002). Descriptive statistics (PROC FREQ) were calculated for each variable to identify frequency distributions. For categorical variables, the percentage response was presented including the number of observations. Additionally, the relationships between categorical variables of interest were studied. For continuous variables, the mean, minimum and maximum values were reported. The associations between farmer responses as well as observed values were tested for normality and examined using Pearson’s chi-squared tests. From here, logistic regression (PROC LOGISTIC) was used to investigate associations between non-normally distributed variables with binary outcomes and categorical variables that had been identified as significant (*p* < 0.05).

## 3. Results

### 3.1. General Herd Information

Spring calving was the predominant system employed (96.1% of farms) with only 3.9% adopting spring-autumn calving systems. On average, 254 cows were expected to calve on-farm in spring 2020 (range: 87 to 550 cows) and nine in autumn (range: 8 to 10 cows). The expected calving season length ranged from 8 to 14.5 weeks (median: 11.6 weeks). Farmers expected to rear approximately: 68 replacement heifers (range: 0 to 250), seven bulls (range: 0 to 62) and 13 additional heifers (range: 0 to 120) past the point of weaning. The destination of calves not reared on-farm are explained later in the results section.

### 3.2. Farm Facilities: Space Allowance and Cubic Air Capacity

During visit 2, which coincided with peak calving, 41.2% of all farms surveyed (N = 51) had deviated from the calf housing plan outlined ahead of calving commencement. Of farms who deviated from the housing plan (visit one), 17.7% used more houses and 23.5% used less. When asked if at least one house was overcrowded on-farm out of all houses they declared, 54.9% said yes and 45.1% said no. However, when houses were examined for space allowances of 1.5 m^2^, 1.7 m^2^, and 2.0 m^2^ per calf, at least one house was overcrowded on 20%, 33%, and 49% of farms, respectively.

Based on the total number of houses declared by farmers (N = 137), 32.9% of these were thought to be overcrowded by farmers. Examination of occupied calf houses (N = 121) for space allowances of 1.5 m^2^, 1.7 m^2^, and 2.0 m^2^ per calf showed that 9.9%, 18.2%, and 27.3% of all occupied houses were overcrowded, respectively.

Group sizes of ≤12 were found in 71.6% of all calf groupings (N = 394) across farms. A cubic air capacity of at least 7 m^3^ per calf was provided in 95.9% of all calf houses evaluated (N = 121). In 33.1% of all calf houses examined (N = 121), airspace was shared with older animals. Farmers whose houses had sufficient space for calves (space allowance and cubic air capacity; OR = 0.243; CI = 0.08 to 0.77) or had larger herds (OR = 0.497; CI = 0.296 to 0.837) were less likely to have issues with diarrhoea in the calves as perceived by the managers (based on farmer perception of diarrhoea issues, responses were yes, no, not particularly).

### 3.3. Farm Facilities: House Structures and Facilities

Individual pens for new-born calves were 0.8 m wide by 1.25 m long (minimum) in 85% of houses using such pens (N = 35). Out of the houses whose individual pens did not provide this area to calves (N = 5), pen length was shorter in 20.0% of cases, and width in 80%. Front only perforations were provided in 48.5% of individual pens, and 24.2% provided side only perforations (27.3% had both). A group pen floor slope of a 1:20 (2.86°) fall was not found in 86.3% of calf group pens evaluated (N = 394). The median slope in all calf pens was 1.6° (range: 0 to 8.16°). Partitions between pens were at least 1.2 m high across 67.4% of pens where divisions were applicable (N = 193) (excl., solid wall divisions from floor to roof). A cold water supply (i.e., tap or water faucet), to facilitate the provision of water for calf and human (to facilitate labour efficiency during water provision), was not provided in 31.6% of sheds surveyed. Wind speeds greater than 0.5 m/s at calf level were present on 3.3% (4/121) of calf sheds surveyed. Relating to light intensity, a minimum level of 50-LUX was provided in 70.3% of all houses evaluated (N = 121). Average house temperature (indoor) was 10.6 °C (range: 5.7–17.2 °C), with average external temperatures of 11.1 °C (range: 3.4–20.9 °C). Average relative humidity in-house was 73.0% (range: 47.5–100%) and the average outdoor humidity was 73.0% (range: 31.6–100%).

### 3.4. Farmer Opinions

The majority of farmers rated welfare on their farm as very good (72.6%), they also rated their calf husbandry skills (82.4%) or the main rearer’s husbandry skills (70.8%) as very good (Table 1). The majority of farmers (58.8%) said they were unconcerned about the upcoming spring workload. While farmers with the largest herd sizes had a range of concerns from being somewhat to very concerned about the workload (62.5%). Retaining non-replacement calves for >10 days (current regulation; DAFM 2007) in spring was not a concern for the majority of farmers (64.7%), with 45.1% reporting to be extremely or very prepared if this happened. Farms with large herd sizes were also very unconcerned about keeping non-replacement calves (37.5%) and were very prepared if this was required (62.5%). Farms with the smallest herd size were not concerned about keeping non-replacement calves longer than 10 days (40%; 4/10), but these farms were somewhat to very unprepared if this happened to occur (60%; 6/10).

### 3.5. Calf Housing and Grouping

Calving and calf rearing pens were in different houses on 88.2% of all farms (N = 51). For these, the mean distance between calving and calf rearing pens was 59.1 m (range: three to 500 m; any additional calf house that required transit by car was not included in this metric due to distance inaccuracies compared to the trundle wheel). Multiple houses were used to rear calves on 84.3% of all farms (N = 51; range: one to seven; Supplementary File S1). The basis for allocating calves to different houses varied, but calf sex was the most common separation criterion (42% of farms; N = 51), according to farmers. Provided information also indicated that grouping of calves directly post-birth, rather than using individual pens, was common practice among farms (58.8%; N = 51). Additionally, the majority of farms kept heifer and bull calves in different pens (76.9%; N = 51).

Age was the most common basis for separation of calves between pens (37%; Table 2). Heifer calves were more likely to be in fixed pens for the duration of the rearing period while bull calves were more likely to be in dynamic groups, according to farmers. Bull calves were mainly grouped in groups of 15 or less (66.0% of farms; N = 51) while the majority of heifers were penned in groups of 16 or more (56.9% of farms; Table 2). Most farms rear calves in both indoor and outdoor environments (62.8%; N = 51), with movement outdoors common before weaning (76.5%). Farmers who did not separate bull and heifer calves (12/51; n/N) were more likely to move calves outdoors before weaning (OR = 40.54; CI = 2.69 to 610.22). Outdoor exposure typically commenced after a calf was three weeks old (82.4%; N = 34; i.e., 34 farms move calves outdoors after 3 weeks old).

### 3.6. House Structures, Modifications, and Investments

Since 2016 cow numbers have increased on 88.2% of farms surveyed (N = 51), with the median level of expansion being 22.2% (range: 0 to 57.5%), relative to pre-expansion herd size. Of respondents who expanded (N = 45), 75% stated calf housing increased to account for expansion. The average age of calf housing was 29.5 years (range: one to 100 years), however according to farmers surveyed (N = 51), 51% built a calf house in the past 10 years. If no new calf house was built in the past 10 years (25/51), farms were less likely to have a purpose-built house (OR = 0.10; CI = 0.01 to 0.79). According to farmers, the number of calves a house was designed to hold ranged from 8 to 200 calves. Out of houses declared (N = 137), 36.5% were purpose-built for rearing calves, however 78.4% of farms (N = 51) had at least one calf house which was purpose-built for calf rearing. Of the houses not specifically built for calf rearing, they were most commonly used for cow and cattle housing, followed by straw storage, among others (Table 3). Modifications to improve suitability for rearing calves were made to at least one house on 76.5% of farms, 83.7% of which were made in the past 10 years. Half of the modifications were to reduce labour, followed by improving calf health (41.9%) and safety (8.1%; animal handling provisions). There were plans to invest in calf rearing facilities on 51% of farms, the majority of which would be made within the year (57.7%; N = 26). Sources for information regarding investments vary among farms. Alternative housing, such as calf hutches and igloos, are not used on 92.2% of farms (N = 51), however 41.3% of farmers would consider using them in the future.

### 3.7. Calf Rearing Labour Units

Additional labour units were hired on 84.3% of farms (N = 51), with the majority hiring one person (51.2%; Table 4). Additional labour units were hired specifically for the calving season on 54.9% of farms, with most hiring one other person (96.4%) and a few hiring two people (3.6%). Common methods for sourcing additional labour units were students (25.8%), word of mouth (25.8%) and farm relief services (13.6%), among others. Over 72% of farms have two to three people involved with calf rearing. The average number of calves per labour unit was 98, (range: 36 to 250). Larger herd sizes (201 to >300 cows) typically had 51 to 150 calves per labour unit (86.2% of large farms; 25/29), whereas smaller herd sizes had 36 to 100 calves per labour unit (86.4% of small farms; 19/22). When more than one person is involved, overlapping in the handover of duties occurs on 87.8% of farms (N = 51), according to farmer interviews. Additionally, a calf rearing guide is available to all people involved on 73% of farms (proof of document not requested during survey). All primary calf rearers were present on the farm more than 5 days per week (100%) and commonly have >20 years of experience with calf husbandry (64.7%).

### 3.8. Calf Feeding

In general, frequency distributions varied relating to colostrum, transition milk and daily feeding of bull and heifer calves (Table 5). Regardless of sex, most farms (N = 51) stated they fed calves within two hours of birth (80.4%) using a bottle and teat, or stomach tube if not drinking (40.0%). Calves were typically fed two to three litres of their own dam’s first milk as their first feed. According to farmers, colostrum was stored on 72.6% of farms, with the main storage location being the fridge (43.5%). Quality was tested on 31.4% of farms, with 75% (12/16) testing all colostrum samples. Refractometers were the most commonly used implement (93.7%; 15/16) to test quality (6.3% used colostrometers). All calves were fed six or more feeds of transition milk most often. Following this, bulls were mainly offered whole milk (50%), while heifers were predominantly offered milk replacer (69.5%); however, bull calves were more likely to be fed waste milk (high somatic cell count/antibiotic) than heifers. Post-colostrum milk was typically fed using multi-teat feeders (non-compartmentalised). Of farms that switch from twice-a-day to once-a-day feeding (41.2%), 42.9% of these farmers did so before a calf was three weeks old.

According to farmers, calves were weaned gradually on 91.7% of farms (N = 48; any unsure or withheld number of days for weaning were omitted from dataset; three farmers). However, determined by the number of weaning days (<4 days being abrupt), 81.3% of farms weaned calves gradually. Farmers generally used a combination of methods (54.9%; N = 51) to assess if calves were ready to be weaned, the most common method was visual assessment of readiness (66.7%), followed by age (37.3%), weight (35.3%) and concentrates (23.5%; all N = 51).

### 3.9. Hygiene and Cleaning

Feeding equipment was washed daily on 84.3% of farms (N = 51), according to farmer responses, with a cleaning agent used at least weekly on 56.9% of farms (at least monthly on 64.7% of farms). A hose was used on 94.1% of farms to clean feeding equipment and facilities, with 47.9% of these farms reporting hose water pressure as high (39.6% medium; 12.5% low). Pens were cleaned out fortnightly, at least, on 54.9% of farms, however regardless of frequency, 76.5% of farms used a disinfection agent when cleaning out occurred. Calves moved outside the pen when cleaning took place on 41.2% of farms. Of houses declared, farmers believe 32.1% could have a better layout to facilitate cleaning.

### 3.10. Calf Health

According to farmers, diarrhoea was the most prevalent calf related health issue, followed by pneumonia and navel infections (Table 6). The average calf morbidity on farms in 2019 (based on previous year recall), as reported by farmers, was 7.5% with a range of 0.9 to 28.8%. Veterinary assistance was not required for calves on 21.6% of farms in spring 2019 (N = 51), with 49% of farms requiring assistance one to two times (including issues related to births). According to farmers, health issues arose on-farm as a result of overcrowding on 21.6% of farms, where diarrhoea was the main illness indicator for these farmers (50%). Forty four percent of farmers reported diarrhoea related problems on-farm mentioning *Cryptosporidium* and Rotavirus as the most frequent causative agents (58.6%). Pneumonia was cited as an issue on 15.7% of farms, particularly when a calf was two to three weeks old (44.4% of farms). Average calf morbidity up to the point of visiting in 2020 (calf requiring antibiotic treatment; expressed as percentage of total cows calved at point of visit) was 4.2% (range: 0 to 80%). The average calf mortality up to the point of visiting in 2020 (expressed as percentage of total cows calved at point of visit) was 3.6% (range: 0 to 9%).

### 3.11. Destination of Surplus Calves

When managing surplus calves the previous year (2019), 80.4% of farmers used multiple avenues when moving these animals off-farm. Calves were most often sold to a buyer (47.9%), followed by the mart/auction (32.3%) and export (19.8%). In 2020, of the farmers who had moved surplus calves off-farm (90%; N = 51), sourcing a buyer was most common (49.6%), followed by mart (31.8%) and export (18.0%).

## 4. Discussion

### 4.1. Facilities

The majority of farms surveyed maintained their original calf housing plan during peak calving in spring (i.e., same number of houses used in spring as specified pre-calving). This indicates farmers had good foresight and planned effectively for spring expectations. Additionally, some farmers used less houses than expected, further suggesting heightened readiness for various outcomes in spring. Over half of farmers said at least one calf house was overcrowded, however when space allowance was evaluated on-farm, most houses were not. This is evident at the 1.5 m^2^/calf space allowance cut-off, a legal requirement for all calves in Ireland. This indicates farmers were self-critical in relation to overcrowding on-farm and may overestimate overcrowding issues, which may be beneficial for calves in terms of their welfare. Current space recommendations of 1.5 m^2^/calf reflect a live weight of less than 150 kg (EU regulations: Council Directive 2008/119/EC), increasing thereafter. However, this study indicates most calves move outdoors pre-weaning, meaning calves would not remain indoors to achieve such weights. Therefore, movement of calves below 150 kg outdoors at an early stage may facilitate space allowances that move toward the lower end of the regulatory scale, in these situations. That said, space allowance is one of many management decisions made at farm level which influences calf welfare, such as group sizes [11] and sharing airspace [22], among others [23]. While most farmers said group sizes were often > 12 calves/pen, spring examination indicated groupings were most often < 12 calves/pen. This is positive, as smaller calf groupings (ideally < 10 calves) is linked to reduced respiratory related issues [11] and improved welfare (expression of play behaviour; [6]). Larger groupings likely coincide with automatic milk feeder usage [24], which also has some welfare benefits [25]. Sufficient air volume was commonly provided to calves on-farm which is positive for calf health. The promotion of air changes within a house regulates temperature and humidity while minimising stagnation of airborne microorganisms [26]. Similar to a previous study [27], an association was found in this study whereby diarrhoea was less likely to be an issue on-farm if sufficient air and floor space allowances were provided to calves. Airspace is not often shared with older livestock and calving pens are most frequently in a different house to the calf-rearing house. Older livestock tend to carry and transfer pathogens to young stock which can threaten calf health, particularly in relation to respiratory infections [28].

Sheds utilising individual pens generally meet the legal minimum specifications (0.8 m width by 1.25 m length; DFAM 2016), however most do not provide both front and side contact with adjacent calves. Given time spent in these pens does not generally exceed five days (grouping occurs thereafter), social isolation may not be a considerable issue during this short timeframe (in natural settings, calves commence group socialisation and increase distance from dam in second week of life; [29]). That said, most farms group calves immediately post-birth, meaning this is not an issue. Grouping post-birth can save labour as personnel can care for calves collectively rather than individually. It can also lessen equipment requirements (i.e., group feeders rather than both individual and group feeders). Most group pens did not have a sufficient 1:20 slope, likely lending to drainage issues [30]. This can cause excrement accumulation within pen, saturating bedding and thus increasing risks of calf illness [8]. The majority of houses did not have any draughts (defined as a wind speed greater than 0.5 m/s at calf level; [31]) however, for those that did, most were not at calf level. As five to six air changes are expected within a house every hour [14], this overhead air movement may promote these changes. Airflow is cited as a way to safeguard calf health in terms of regulating temperature, humidity, gases (e.g., ammonia) and stagnant microorganisms, which is beneficial to overall welfare [26].

### 4.2. Calf Grouping and Movement Outdoors

Two calf houses were commonly seen across farms, which was somewhat likely due to the main separation criterion for different houses being sex. This may be a biosecurity precaution to minimise calf exposure to buyers purchasing calves on-farm, who may introduce foreign pathogens to a house which was a concern brought forward by farmers from a study by Wilson et al. [32]. Additionally, more than one house would allow for biocontainment of disease outbreaks among all calves [33], by limiting issues to one house. If houses were not separated by sex, most farmers kept bulls and heifers in separate pens. This is favourable because it would mean calf groupings would be more static than dynamic (i.e., heifers not continually disturbed due to sale of bull calves). Dynamic groups are groups where new calves are continuously introduced and removed, and can have negative implications on calf health, growth, and overall welfare ([34]; social stress exhibited in five-month-old dairy heifers [35]). Heifer calves were often maintained in fixed pens with calves of similar ages, meaning once a group pen became full, no calves were moved in or out of the group. This grouping minimises pathogen exposure as well as facilitation of disinfection between group pens.

A fusion of indoor and outdoor calf rearing is popular among farmers. For the calf, outdoor exposure pre-weaning allows for the introduction of grass as a feed source, thus potentially improving foraging skills. Research indicates that sheep forage more efficiently when they experience plants previously [36], which may be the case for calves also. Additionally, as the season progresses, calf houses with organic material offer the perfect environment for pathogen proliferation (warm, damp, and humid conditions) and infections can spread exponentially among confined calves [22]. Movement outdoors may alleviate space allowance and stocking pressures within the shed, reducing the associated risks. This would directly impact calf rearing labour, as tending to sick calves is likely a very laborious process (similar to time consuming process of providing individual health care to poultry; [37]). Calves typically moved outdoors after they reach three weeks old; this delay can be positive because calves become more robust with age, i.e., improved thermoregulation (older calves have a better ability to thermoregulate; [38]) and digestive development (movement towards functioning ruminant; rumen fermentation may aid with heat production; [39]). Additionally, shelter is always provided to calves, facilitating micro-climate creation, warmth via body heat and protection from interchangeable spring weather patterns [40,41].

### 4.3. Structure Modifications and Investment

In general, farmers said housing had increased in response to on-farm expansion. When examined, sufficient space allowances among calves indicate these sentiments to be true. This is positive because, it is evident facilities have grown to consider cow needs, but it was unclear if this had also occurred for calves. Furthermore, this echoes findings from a previous study in Ireland [6], where farms also had sufficient calf space allowances. While calf house age varies, half of farms built a purpose-built calf house in the last 10 years. In contrast, farms were less likely to have purpose-built facilities if a house was not built in the last 10 years, which suggests conversions of existing sheds occurred. However, while conversions are often necessary, it is important to ensure these spaces are appropriate for both calf and carer needs [9]. For example, when considering water provisions, facilities that promote efficiency (i.e., water source within the house) should be considered. Measures such as this have taken priority for surveyed farmers, as most made modifications to at least one house in the past 10 years for labour gains and calf health. This indicates that farmers heeded advice regarding increasing labour productivity to facilitate herd expansion post-quota abolition [42]. Investment in rearing facilities within the year is a priority for half of farmers. Most plan to seek advice from multiple outlets (e.g., advisor, builder etc.), however out of the 50% of farmers who will draw on their own knowledge, over half will not consult another party before investing. While farmer knowledge is extremely valuable for on-farm investment decisions, the search for up to date and relevant research and information from others should also be encouraged. A recent study has shown the value of farm visits, walks and group meetings as a less intimidating space to share knowledge selectively and give contextualisation for farmers about aspects of other farms [43]. Effective use of discussion groups in this manner could be beneficial encouraging collaboration during farm building investment. For example, most farms surveyed did not use alternative housing options such as calf hutches but, over one third of farms would consider them in the future. This highlights a potential avenue for future research detailing how alternative housing systems might be integrated into an Irish system and whether it might be an alternative investment to permanent housing.

### 4.4. Labour at Calving

Additional labour is typically hired on-farm, suggesting a slight redirection from what has been typically seen on European farms, whereby half of working labour is accounted for by family [44] The average number of calves per rearer was 98, similar to what is recommended for cow to labour unit ratio (100:1; [45]). While there is no specific ratio for this relative to calves, it is likely farmers have adopted a similar approach to cow recommendations. The hiring of additional labour units for calving is an expected response to what is highlighted as an extremely labour-intensive period [46]. However, due to seasonality of much farm work, it is often unattractive for employees [47]. In response to this challenge, farms appear to frequently utilise students, who offer short-term solutions, to bridge this labour gap. Most farms have a calf rearing team of many people, however in this case careful and clear communication is required to ensure rearing runs smoothly. This echoes a farmer sentiment in a recent study [48] which noted the difficulty in identifying inconsistencies in calf care when too many people are involved in the process. Most farms demonstrate good communication, whereby time is taken to hand over daily tasks from one rearer to another. Additionally, calf rearing guides detailing standard operating procedures are available on most farms aiding with role transition and task completion. These are a tool, which has been cited as valuable in the calf rearing process on-farm [48]. All primary calf rearers are on-farm at least five days per week. While this provides consistency within the rearing process, burnout is a risk when a person operates in stressful conditions for long periods of time [49]. Studies indicate people are less productive and more inclined to make work related mistakes if over-worked [50].

### 4.5. Feeding, Hygiene and Cleaning

According to farmer responses, colostrum feeding practices on-farm are in line with recommended advice, whereby colostrum is fed to calves within two hours of birth [14]. On-farm improvement can be made in relation to colostrum volume fed to calves: currently typically 2–3 L, whereas the aim is for calves to consume at least 3 L [14]. Additionally, while colostrum testing appears to have increased on-farm since a survey carried out on Irish farms in 2017 [51], improvements could still be made. Considering colostrum is a calf’s first line of defence against infection, it is extremely important to ensure that colostrum provided is of high quality (high quality means >50 g/L IgG; [52]) to facilitate appropriate passive transfer [53]. Few farms on the study facilitated cow calf contact in the form of sucking post-calving (colostrum consumption by suckling dam), with no farm engaging in this practice following colostrum consumption. This would indicate that cow calf contact is not common on Munster dairy Farms in Ireland. Calves are typically offered upwards of five feeds of transition milk following colostrum, a finding similar to previous research conducted on Irish dairy farms [6]. While according to farmers surveyed, waste milk is offered more frequently to bulls than heifers, lower numbers of farmers were engaging in this practice compared to a previous Irish study [6]. This is a positive indicator that information campaigns (AHI) related to this area are proving successful, as it is strongly advised not to feed waste milk to calves on-farm, as this is linked to antimicrobial resistance on-farm [54]. Whole milk is most commonly fed to bulls, possibly for economic reasons relative to the cost of milk replacer, while milk replacer is most commonly fed to heifers, potentially for biosecurity and disease transfer reasons or to offset reductions in cash flow due to a reduced volume of milk sales. Asheim et al., [55], compared the reduction in saleable milk with cow-contact systems. Milk is mostly fed manually via non-compartmentalised teat feeders, thus there is no control over the quantity calf milk ingested in a group setting. This is concerning, particularly in a restricted feeding capacity, because drinking speeds vary considerably among calves (with sorting by drinking speed common in the veal sector; [56]). Without sorting due to drinking speed, calves with fast drinking speeds would consume significantly more milk than calves which drink more slowly. Although automatic feeders have been noted as useful technology in relation to calf rearing [57], a relatively low uptake of this equipment was found on-farm. Manual feeding systems were also more popular than automated systems in a Canadian study, whereby farmers found costs a barrier to investment in automatic feeders, and instead liked the level of disease detection and ease of calf handling associated with manual systems [57]. While not surveyed directly on reasoning, it is possible this may also be the case for the farmers in this survey. Another point of concern related to feeding practices is the age once-a-day feeding commences. Almost half (42.9%) of farms implement once-a-day feeding before three weeks old, which is a welfare concern. Once-a-day feeding should only commence once a calf reaches 28 days old as their digestive systems are unable to manage infrequent high volumes of feed before this point [14], however, this is still quite a young age. Furthermore, this infringes on a calf’s ability to perform natural suckling behaviours, in a way that would mimic a natural scenario with the cow (a highlighted benefit of automatic milk feeders [57]).

The majority of farms wash feeding equipment daily with over half using cleaning agents at least weekly (some more frequent). Inadequate and infrequent cleaning is a huge issue in calf rearing; hygiene management (as well as biosecurity measures) can minimise infectious agent transmission among calves [33,51,58]. Half of farms surveyed clean out pens fortnightly, with the majority also using disinfection agents. Appropriate hygiene and environmental disinfection are an effective way to control the spread of disease agents [59,60]. Farmers have highlighted almost one third of sheds declared as needing modifications for labour efficiencies around cleaning out. This demonstrates the ability to look at the farm critically and identify areas requiring improvement. Critical thinking is an important skill to attain in the workplace as it demonstrates communication, decision-making, analytical and problem-solving skill development [61], all which likely lends itself to the development of viable farm practices.

### 4.6. Health and Calf Movement

The most common calf health issues, as experienced by the farmers in this survey, were diarrhoea and pneumonia. This finding is in line with literature which states these areas as problematic for calf health [62,63]. In relation to diarrhoea, *Cryptosporidium* and Rotavirus were mentioned as the two main causative agents, among others. This is in accordance with much other calf research (e.g., [64,65]. According to farmer opinion, health issues related to overcrowding were not experienced on most farms. However, for farmers that believed this was an issue on their farm, health complications due to overcrowding generally presented as calf diarrhoea (an eventuality previously outlined by Bazeley [66]). Mortality statistics reported by farmers for 2019 were quite similar to a previous study conducted on Irish farms in 2017 [6], however it must be remembered that in this instance, mortality statistics were recounted by farmers (recounted in December 2019 for calves born in 2019).

Farmers utilise multiple means to move surplus calves off-farm, however in 2019 and 2020, sourcing a buyer was the most popular method. Compared to export, a buyer minimises the degree of transport these calves are subjected to and ensures calves move regularly off-farm creating more space for those remaining. Transport is stressful for calves and distance travelled has been linked to calf morbidity [67] and mortality during or shortly after transportation has occurred [68]. Additionally, when calves are sold at marts (auctions) co-mingling with calves of other dairy herds occurs [69], which can increase the risk of pathogen exposure.

### 4.7. Farmer Opinions

Most farmers surveyed rated their calf welfare and calf husbandry skills as ‘very good’. This result is positive, as most farmers acknowledge that improvements can be made to their skills, and it perhaps demonstrates an openness to upskilling in farm related practices (due to the top rating not being selected frequently). A similar willingness of farmers to engage in various levels of upskilling was found in New Zealand [70], however this article highlighted the need to provide appropriate supports to the farmer to do this. Animal welfare has been acknowledged as dynamic and changing constantly [71], as a result continued professional development and upskilling is important to ensure practices and knowledge are in line with welfare concepts (e.g., One Welfare; [72]). Conversely, farmers rating themselves as ‘very good’ in terms of their calf welfare and husbandry may also suggest they over-rate their qualities in these areas. While many may have knowledge related to a number of welfare issues, they may be blind to issues related to their own farm.

### 4.8. Study Limitations

It is important to recognise limitations that exist within this study in relation to herd selection and farmer response bias. The farmers surveyed in this current study may not be typical of all dairy producers in Ireland for a number of reasons including larger average herd size, interaction and participation in discussion groups, as well as a high level of data recording carried out on-farm compared to the national average. That said, due to the geographical location of the farms surveyed (the region in Ireland most densely populated with dairy cows) as well as the larger nature of herd sizes, it could be argued that these farmers are also those who may be most likely to remain in dairying and represent the future of the Irish dairy herd/producer. It also must be recognized that the study population are not representative of the whole Republic of Ireland, but rather the Munster region, which accounts for 56% of dairy cows in the country.

A potential social desirability bias may also have existed, whereby survey participants adjust their answers to reflect what they perceive researchers would find acceptable (as mentioned [57]), particularly related to the face-to-face nature of data collection. However, care was taken to communicate that survey responses were in line with GDPR guidelines, meaning all information collected was anonymous and confidential, with no identifiable farm or farmer characteristics report thereafter.

## 5. Conclusions

To conclude, farmers demonstrated sufficient planning for spring, providing appropriate housing and space allowances for calves that facilitated group housing post-birth, influencing calf welfare positively. Planning was also apparent by the lack of concern among farmers regarding upcoming spring workload. Large proportions of sheds are converted for calf housing, so emphasis must be placed on modifying sheds appropriately so provisions are made to safeguard animal and farmer welfare (e.g., drainage and slopes and facilitate cleaning out). Management components of rearing systems appear in line with current sectoral recommendations (e.g., advice against feeding waste milk), and improvements have been made, however areas require further attention on many farms (e.g., colostrum testing and extended working hours), to safeguard calf welfare and reduce associated workloads. Therefore, calf housing provisions seem to be sufficient for calf numbers born on-farm in the Munster region in Ireland, however structural components and management decisions should be continually reviewed to ensure that calf and human welfare is considered.

## Figures and Tables

**Table 1:** Farmer opinions (N = 51) on calf related topics.

Variable	Response	Percentage
Rate calf welfare on-farm	Extremely poor Very poor Somewhat poor Somewhat good Very good Extremely good	0 0 0 17.7 72.6 9.8
Rate your calf husbandry skills on-farm	Extremely poor Very poor Somewhat poor Somewhat good Very good Extremely good	0 0 2.0 13.7 82.4 2.0
Rate calf husbandry skills of main rearer on-farm	Extremely poor Very poor Somewhat poor Somewhat good Very good Extremely good	0 0 0 29.2 70.8 0
Concerned about associated workload with upcoming calving	Extremely unconcerned Very unconcerned Somewhat unconcerned Somewhat concerned Very concerned Extremely concerned	7.8 29.4 21.6 29.4 7.8 3.9
Concerned about keeping non-replacement calves for >10 days in spring	Extremely unconcerned Very unconcerned Somewhat unconcerned Somewhat concerned Very concerned Extremely concerned	9.8 25.5 29.4 15.7 13.7 5.9
If had to keep non-replacement calves for >10 days how prepared are you	Extremely prepared Very prepared Somewhat prepared Somewhat unprepared Very unprepared Extremely unprepared	5.9 39.2 21.6 21.6 11.8 0

**Table 2:** Calf pre-weaning rearing location and grouping information (N = 51 unless otherwise stated).

Variable	Response	Percentage
Use individual pens	No Yes	58.8 41.2
Time in individual pens post-calving (Bull N = 20; Heifer N = 21)	**Bulls** 1–2 days ≥2–5 days **Heifers** 1–2 days 2–5 days	45.0 55.0 42.9 57.1
Most common separation criterion for calves between pens (N = 92)	Age Breed Drinking ability Sex Size	37.0 2.2 12.0 28.3 20.5
More than one selection criteria for separating calves between pens	Yes No	70.6 29.4
Bulls and heifers grouped in separate pens	Yes No	76.9 23.1
Formation of calf groups	**Bull** All-in all-out Subject to change **Heifers** All-in all-out Subject to change	25.5 74.5 54.9 45.1
Calf group size	**Bulls** 1 to 10 11 to 15 16 to 20 21 to 25 >25 **Heifers** 1 to 10 11 to 15 16 to 20 21 to 25 >25 Unsure	46.0 20.0 16.0 4.0 14.0 25.5 15.7 11.8 9.8 35.3 2.0
Calves rearing location pre-weaning	Indoor Indoor & outdoor	37.3 62.7
Age calves begin moving outdoors (N = 32)	0–3 weeks 4–8 weeks ≥8 weeks	17.7 58.8 23.5
Type of outdoor facility (N = 32)	House with field access Field with shelter Both	60.6 30.3 9.1
Time calf remained indoors post-wean	Outdoors before wean <2 days 3–5 days 6–10 days >10 days Unsure	58.8 11.8 7.8 9.8 9.8 2.0

**Table 3:** Summary of house structures, modifications, and investments of surveyed farms (N = 51).

Variable	Response	Percentage
Previous use of houses not purpose built for calf rearing (N = 137)	Calving Cow/Cattle housing Feed House Machinery Parlour Pigs/Sheep Potato Straw General/Other/Unsure	10.3 32.2 4.6 1.2 4.6 6.9 4.6 1.2 28.7 5.8
Plan to invest in calf rearing facilities (N = 51)	Yes No Unsure	51.0 47.1 1.9
When will investments be made (N = 26)	<1 year 2–5 years 6–10 years	57.7 38.5 3.8
What investment will be made (N = 26)	Converting old shed Extension onto house New house Roof Feeding equipment Pen structures	3.1 12.5 40.6 3.1 18.8 21.9
Source likely to seek advice from related to investment (N = 26)	Advisor Builder Company Discussion Self Specialist	19.4 5.6 5.6 11.1 50.0 8.3
Plan to consult other party (except self) for investment advice (N = 26)	Yes No	46.2 53.9

**Table 4:** Labour unit related information for the surveyed farms (N = 51 unless otherwise stated).

Variable	Response	Percentage
Number of labour units hired on-farm (N = 43)	1 2 3	51.2 30.2 18.6
Method of sourcing additional labour units (N = 66)	Employees Farm relief services Family Internet Word of mouth Neighbour Students Newspaper	1.5 13.6 9.1 4.6 24.2 12.1 25.8 9.1
Number of people involved with calf rearing on-farm	1 2 3 4 5 6	5.9 39.2 33.3 15.7 3.9 2.0
Calf husbandry experience of main rearer (years)	1–5 >5–10 >10–15 >15–20 >20	11.8 7.8 7.8 7.8 64.7

**Table 5:** Colostrum, transition, and general feeding practices for the surveyed farms (N = 51 unless otherwise stated).

Variable	Response	Percentage Bull *	Percentage Heifer *
Latency from birth to first feed (hours)	≤2 ≤2 if possible ≤6	80.4 11.8 7.8	
Milk provided for first feed	Own dam’s first milk Another cows first milk Pooled first milk Own dam or another cows first milk Own dam or pooled first milk Another cow or pooled first milk	29.4 25.5 27.5 9.8 3.9 3.9	37.3 23.5 21.6 9.8 3.9 3.9
Volume of milk given as colostrum	<2 litres 2–3 litres >3–4 litres >4 litres	0 58.0 40.0 2.0	0 56.0 42.0 2.0
Method used to feed colostrum	Bottle and teat Stomach tube only Bottle and teat or stomach tube if wont drink Left with cow Left with cow or bottle and teat Bucket and teat	9.8 27.5 49.0 5.9 2.0 5.9	
Location of choice for colostrum storage (N = 69)	Freezer Fridge Room temperature	31.9 43.5 24.6	
Number of transition milk feeds given	0 1–2 >2–3 >3–4 >4–5 ≥6	0.0 9.8 17.7 11.8 21.6 39.2	2.0 9.8 15.7 13.7 25.5 33.3
Method used to feed transition milk	Buckets (no teats) Compartmentalised multi-teat feeders Individual buckets and teats Multi-teat feeders (non-compartmentalised)	1.7 16.7 16.7 65.0	
Type of milk fed following transition milk	Milk replacer Waste-milk antibiotic Waste-milk high SCC Waste-milk both Whole milk	15.5 12.1 5.2 17.2 50.0	69.5 1.7 1.7 3.4 23.7
Method used to feed milk following transition feeding	Compartmentalised multi-teat feeders Automatic feeders Individual buckets and teats Mobile multi-teat feeders Multi-teat feeders (non-compartmentalised)	14.3 5.4 1.8 8.9 69.7	9.7 12.9 0 21.0 56.5
Volume of milk fed per day (litres)	4 >4–5 >5–6 >6–7 >7–8 >8–10 Adlib	11.8 19.6 54.9 5.9 5.9 0 2.0	9.8 15.7 60.8 0 3.9 7.8 2.0
Manual feeding: feeders shared between pens	Yes No	90.2 9.8	
Manual feeding: feeders washed before sharing (N = 47)	Yes No	10.6 89.4	
Frequency of milk feeds per day	Twice Twice then once-a-day Many (automatic feeder)	52.9 41.2 5.9	
Calves fed at same time every day	Yes No	100 0	
Commencement of once-a-day feeding (weeks; N = 21)	<2.5 ≤3 >3–4 >4–5 >5–6 >6	4.8 38.1 33.3 9.5 9.5 4.8	
Calf milk temperature	Warm only Cold only Warm or cold	80.4 9.8 9.8	

* data merged and centred if frequency distributions were identical between bulls and heifers for a variable.

**Table 6:** Summary of farm calf health data based on producer reporting (N = 51 unless otherwise stated).

Variable	Response	Percentage
Main health issues encountered on-farm by calves (N = 78)	Colic Genetic issues Navel infections Pneumonia Diarrhoea	1.3 2.6 14.1 28.2 53.9
Number of calves treated with antibiotics (Spring 2019)	≤10 >10–20 >20–30 >30–40 >40–50 >81	51.0 23.5 11.8 5.9 3.9 3.9
Number of veterinary visits to calves (Spring 2019)	0 1 2 3 4 5+	21.6 13.7 35.3 9.8 13.7 5.9
Overcrowding impact on calf health (N = 10)	Diarrhoea Pneumonia Both	50.0 30.0 20.0
Calf diarrhoea issue on-farm	Yes No Not really	44.0 42.0 14.0
Age diarrhoea becomes issue on-farm (weeks; N = 29)	≤1 >1–2 >2–3 >3–4 Mid-spring Out to grass	17.2 24.1 27.6 20.7 6.9 3.5
Most common calf related diarrhoea encountered on-farm (N = 41)	Coccidiosis Corona *Cryptosporidium* *E. coli* Nutritional Rota Unsure	7.3 2.4 34.2 2.4 14.6 24.4 14.6
Calf pneumonia issue on-farm	Yes No Not really	15.7 66.7 17.7
Age pneumonia becomes issue on-farm (weeks; N = 18)	≤1 >1–2 >2–3 >3–4 >5–6 >7–8 March-born calves	5.6 22.2 44.4 11.1 5.6 5.6 5.6
Location of sick calves (N = 61)	Individual pen Isolation house Isolation pen Remain in group	26.2 6.6 44.3 23.0
Recovered calves go back to original group or enter a new one (N = 45)	Original New Situation dependent	71.1 24.4 4.4

## Data Availability

Data and survey available upon request.

## Supplementary Materials

The following are available online at https://www.mdpi.com/article/10.3390/ani13061019/s1, File S1: Summary of calf housing information.
