# Supplementary material for: A Survey of Calf Housing Facilities Pre-Weaning, Management Practices and Farmer Perceptions of Calf Welfare on Irish Dairy Farms
Source: Animals (Basel). 2023 Mar 10;13(6):1019. doi: 10.3390/ani13061019 (PMC10044077; doi:10.3390/ani13061019)
Supplement: Supplementary file 1 [file animals-13-01019-s001.zip › animals-2210479-supplementary.pdf]

Supplementary files

Supplementary File S1. Summary of calf housing information.

Variable	Response	Percentage
Number of houses used to rearing calves (N=51)	1	15.7
	2	41.2
	3	15.7
	4	19.6
	≥5	7.8
Most common selection criteria for separation between houses (N=69)	Age	39.1
	Breed	5.8
	Drinking ability	1.5
	Sex	42.0
	Size	11.6
More than one selection criteria for separating calves between houses (N=44)	Yes	52.3
	No	47.7
